# Decoding ribosome complexity: role of ribosomal proteins in cancer and disease

**DOI:** 10.1093/narcan/zcae032

**Published:** 2024-07-23

**Authors:** Pedro Fuentes, Joffrey Pelletier, Antonio Gentilella

**Affiliations:** Laboratory of Cancer Metabolism, ONCOBELL Program, Bellvitge Biomedical Research Institute (IDIBELL), 08908, L'Hospitalet de Llpbregat, Barcelona, Spain; Laboratory of Cancer Metabolism, ONCOBELL Program, Bellvitge Biomedical Research Institute (IDIBELL), 08908, L'Hospitalet de Llpbregat, Barcelona, Spain; Department of Physiological Sciences, Faculty of Medicine and Health Sciences, University of Barcelona, 08908, L’Hospitalet de Llobregat, Barcelona, Spain; Laboratory of Cancer Metabolism, ONCOBELL Program, Bellvitge Biomedical Research Institute (IDIBELL), 08908, L'Hospitalet de Llpbregat, Barcelona, Spain; Department of Biochemistry and Physiology, Faculty of Pharmacy and Food Science, University of Barcelona, 08028, Barcelona, Spain

## Abstract

The ribosome is a remarkably complex machinery, at the interface with diverse cellular functions and processes. Evolutionarily conserved, yet intricately regulated, ribosomes play pivotal roles in decoding genetic information into the synthesis of proteins and in the generation of biomass critical for cellular physiological functions. Recent insights have revealed the existence of ribosome heterogeneity at multiple levels. Such heterogeneity extends to cancer, where aberrant ribosome biogenesis and function contribute to oncogenesis. This led to the emergence of the concept of ‘onco-ribosomes’, specific ribosomal variants with altered structural dynamics, contributing to cancer initiation and progression. Ribosomal proteins (RPs) are involved in many of these alterations, acting as critical factors for the translational reprogramming of cancer cells. In this review article, we highlight the roles of RPs in ribosome biogenesis, how mutations in RPs and their paralogues reshape the translational landscape, driving clonal evolution and therapeutic resistance. Furthermore, we present recent evidence providing new insights into post-translational modifications of RPs, such as ubiquitylation, UFMylation and phosphorylation, and how they regulate ribosome recycling, translational fidelity and cellular stress responses. Understanding the intricate interplay between ribosome complexity, heterogeneity and RP-mediated regulatory mechanisms in pathology offers profound insights into cancer biology and unveils novel therapeutic avenues targeting the translational machinery in cancer.

## Graphical Abstract

**Graphical Abstract:**
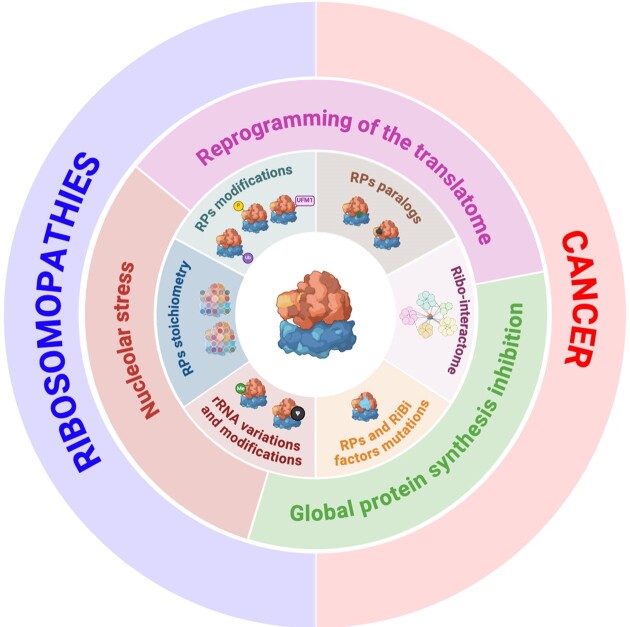

## Introduction

Ribosomes arose in the early stages of evolution as essential molecular machines. Their emergence marked a significant milestone in the proposed central dogma of biology, facilitating the translation of genetic information encoded in nucleic acids into structural and catalytic proteins across all domains of life (1). However, their core structure and function have remained almost unchanged considering the estimated evolutionary time of ∼3 billion years. Despite this apparent structural consistency, the mechanisms that generate new ribosomes are quite divergent between the kingdoms and, as a rule of thumb, their complexity increased during evolution. In this regard, in unicellular and multicellular organisms the replication of cellular biomass, which is a major function of ribosomes, reflects two different needs. Indeed, unicellular organisms, such as bacteria, utilize protein synthesis to replicate the same cell almost indefinitely, thus attacking prokaryotic ribosomes by pharmacological means has revealed an effective strategy against bacterial infections. In multicellular organisms, the complexity of developmental programmes requires a tight regulation of protein synthesis to shape tissues and organs and to maintain their homeostasis. A first level of complexity is provided by the compartmentalization of ribosome biogenesis, which from prokaryotes to eukaryotes has been confined to the nucleolus, involving a fine cross-talk between the nucleus and the cytoplasm. Yeasts have been widely utilized as model organisms to study eukaryotic ribosome biogenesis; however, a remarkable number of regulatory mechanisms appeared in the leap to multicellular life, as demonstrated by the significant number of human ribosome biogenesis-assisting factors not having an orthologue between humans and yeast (2). In this article, we delve into the roles and regulation of ribosomal proteins (RPs) in supporting the growing complexity of ribosomes during evolution, and approach the questions arising on their stoichiometric regulation. Moreover, we tackle the implications of RP alterations in human diseases. We review recent findings establishing how RPs contribute to ribosome heterogeneity, from the discovery of the non-stoichiometric RP composition of ribosomes to the characterization of functional RP post-translational modifications (PTMs). In more detail, we provide an insightful examination of ribosomal proteins and ribosome biogenesis, highlighting their crucial roles in cellular functions and stress responses, from ribosomopathies to cancer. The focus of our analysis underscores the complexity of ribosomal regulation, from evolutionary adaptation to ribosome heterogeneity, offering a detailed understanding of their multifaceted roles in biology and their clinical implications.

## Ribosomal proteins and ribosome biogenesis: from prokaryotes to higher eukaryotes

### The appearance of ribosomal proteins

According to the hypothesis of a primitive ‘RNA world’, life has been shaped around a self-replicating proto-ribosome composed solely of rRNA, which emerged long before the amino acids made their appearance in evolution, alongside the cell as the biological unit of life (3). Several compelling pieces of evidence have sustained the role of catalytic RNA as a self-sufficient molecular entity capable of carrying out enzymatic reactions for peptide bond formation. Recent advancements in synthetic biology have demonstrated the reconstruction *in vitro* of the minimal RNA core element of the ribosome capable of catalysing peptide bonds without the assistance of any RP (4).

This raises questions regarding why RPs have been evolutionarily selected to complement the rRNA functions. One explanation comes from seminal works by the Nomura laboratory supporting the role of RPs in facilitating proper rRNA folding. In an attempt to reconstitute the 30S subunit of *Escherichia coli*, the group demonstrated that in the absence of RPs, rRNA fails to adopt its functional conformation (5,6,7). Consistent with this, a recent study has shown that the co-transcriptional folding of pre-16S rRNA by a hierarchically ordered association of RPs is key to limit the conformational possibilities of the 16S rRNA to a functional three-dimensional structure in a biologically relevant time scale (8). Another rationale for the appearance and conservation of RPs lies in their role in enhancing the translational fidelity of ribosomes and the speed of translation. In this regard, in *E. coli*, a group of mutations clustered under the name of *ram* mutants (*r*ibosome *am*biguity) and characterized by an increased translational error rate, fall in specific segments of not only 16S rRNA but also RP genes (RPGs), as in the case of RPS4, RPS5 and RPS12 (or uS4, uS5 and uS12, respectively, with the univocal nomenclature), thus highlighting the specific role of these RPs in the accuracy of translation [reviewed in (9)]. Furthermore, Lys60 (K60) of RPS12 (uS12 or RPS23 in yeast and humans), a universal RP of the decoding center of the small subunits, is well conserved in all domains of life; however, some hyperthermophilic archaea do show an arginine substitution (K60R) which significantly increased the ribosome accuracy under different stressors, such as high temperature. Introducing the K60R substitution in the eukaryotic homologue of RPS12 in yeast, worms and flies not only increased the translational fidelity but also extended the life span of those organisms, suggesting a link between the loss of proteostasis and the ageing process (10).

Both prokaryotic and eukaryotic ribosomes consist of a small and a large ribosomal subunit. However, the numbers of RPs vary from bacteria, to archaea to eukaryotes, with the mitochondrial and plastid ribosomes being the vestiges of prokaryotic endosymbiosis. To mitigate the ambiguity arising from assigning identical names to ribosomal proteins across distinct species that lack structural and functional similarities, an univocal nomenclature has been proposed (11) which is slowly replacing the old naming system. A set of 33 universal RPs is shared in all the domains of life, suggesting that the ribosome has formed around this core of RPs (12). However a systematic characterization by chromosomal deletion of RPGs in *E. coli* demonstrated that several universal RPs of both the large and the small subunits are dispensable for survival (13). Similarly, a collection of RP deletion mutants in *Saccharomyces cerevisiae* has also demonstrated that 14 RPs are non-essential for survival; however, many of them clustered in the group of eukaryotic-specific RPs (14). A deeper characterization at the molecular level demonstrated that the depletion of almost any of the RPs in yeast leads to a defect in ribosome biogenesis at different steps of the process depending on the RP depleted (15,16) [reviewed in (17)]. These observations suggest that to some extent, certain specific RPs can be compensated by others for ribosome assembly and function, to provide sufficient protein synthetic capacity indispensable for cell survival.

The role of RPs in assisting rRNA folding throughout evolution has been supplemented by an increasing number of ribosome biogenesis factors. While bacterial and archaeal ribosomes can be reconstituted *in vitro*, eukaryotic ribosomes cannot, suggesting a shift towards the involvement of assembly factors in assisting the more complex pathway of production of the 40S and 60S subunits of eukaryotes (12). Prokaryotes have a limited number of assembly factors (∼50 characterized so far) aiding ribosome biogenesis, whereas yeast has >200 ribosome biogenesis factors involved in various steps of rRNA maturation, modifications, nuclear export and quality control of the subunits (18). Furthermore, proteomic analyses of the nucleolus and small interfering RNA (siRNA) screening in human cells have unveiled a plethora of ribosome biogenesis factors with no yeast orthologues, associated with the cell cycle and other cellular functions (2).

### Coordination of ribosomal protein expression and ribosome biogenesis from *E. coli* to *S. cerevisiae*

Ribosomes are essential to sustain basically all cellular activities. While cells can persist without the nucleus, as in the case of human erythrocytes, they cannot survive long without ribosomes. To guarantee the maintenance of the protein synthetic capacity, the cell must prioritize the expression of the transcripts coding for RPs and all ribosomal components, requiring the existence of a hierarchy in the transcriptional program of the cell. Cells from different taxonomic domains have tackled this challenge utilizing different strategies to preserve ribosome production and hence the biosynthetic capacity of the cell.

The remarkable number of players involved in ribosome biogenesis suggests that to be effective, this process must have an organized and coordinated execution. RPs constitute, together with rRNAs, the ‘bricks’ of the ribosomes, and their stoichiometric assembly has posed a challenge concerning the coordination of the transcriptional and translational programs of the cell and their integration with metabolism. In *E. coli*, the 55 RPGs are organized in 19 operons [reviewed in (19)]. If one of the RPs of the operon is produced in excess, it will recognize specific stem–loop conformations at the 5′-untranslated region (UTR), leading to premature transcriptional termination and/or translational repression. Translational regulation, exemplified by the S15 (uS15) operon, involves a mechanism of ‘entrapment’ that secures the ribosome availability to an mRNA whose translation is essential for the biogenesis of ribosomes and can be rapidly turned on when needed (20). Another mechanism that establishes a stoichiometric regulation between RPs and the nascent rRNA is based on competition, with the regulatory RPs of the operons competing for binding on the rRNA or on the cognate mRNA to suppress its expression. It is important to underline that in bacteria, key components involved in the global metabolic activity of the cell, such as the subunits making up the RNA polymerase, are distributed between the RP operons, namely the L10 and the S4/α operons.

The advent of nucleolar compartmentalization of ribosome biogenesis in unicellular eukaryotes has changed the initial paradigm of co-transcriptional ribosome assembly. This evolutionary innovation has introduced multiple regulatory layers, enhancing the process to accommodate diverse metabolic demands, particularly evident in complex multicellular organisms. *Saccharomyces cerevisiae* and *Schizosaccharomyces pombe* have served as model organisms to understand most of the aspects related to eukaryotic ribosome structure, assembly, RPG regulation and integration with cellular metabolism. Comparing those findings with higher eukaryotes, specifically humans, paved the way to our appreciation of what makes us different with respect to ribosome biogenesis, translation and their associated regulation. To produce the 79 RPs of the yeast ribosome, the genome of *S. cerevisiae* is decorated with 138 RPGs, of which 118 are duplicated and 20 are unique genes (21). Thanks to a finely tuned transcriptional regulation at the RPG promoters, this genetic set up guarantees the capacity to produce RPs at equimolar amounts and to coordinate this with the synthesis of rRNA. In this regard, the Shore group has described a sophisticated mechanism of coordination involving the dual action of Ifh1 at the RPG promoters, along with Rap1 and Flh1, and at the CURI complex, where Utp22 can communicate with RNA polymerase I to couple RP and rRNA production. Importantly, this is connected to the metabolic status of the cells through the regulation of these mechanisms by TORC1 and its downstream effector Sch9 (22). However, this regulatory loop has been lost in higher metazoans, that adopted another mode of regulation with the translational tuning of RPs (see below).

### Translational regulation of RPs in human cells

The complexity of multicellular organisms involves the specialization of cell types, which give rise to tissues and organs highly adapted to execute specific functions. This variation of biological responsibilities is mirrored by a tightly controlled metabolic diversification, which in specific cases, such as for haemotapoietic stem cell- (HSC) derived lineages, requires a highly regulated tuning of the protein synthesis rate (23). Indeed, the regulation of the protein synthetic capacity is probably the most sensitive and dynamic cellular adaptation known, intricately linked to the availability of ribosome components, especially RPs. The activation and inactivation of RP mRNA translation plays a pivotal role in this metabolic flexibility, and the translational engagement of RPs is among the first events observed in fertilized oocytes to generate new ribosomes in the zygote (24). This regulation is achieved by an RNA motif termed TOP (tract of polypyrimidine) present at the transcriptional start site (TSS) of basically all RP mRNAs and of many translational factor mRNAs (25,26), which makes them translationally coupled to the status of the mammalian target of rapamycin (mTOR) (27,28,29). Indeed, as a sensor of intra- and extracellular cues, mTOR is crucial to accommodate the cellular responses to the resources available and the signals received from the microenvironment. Due to the high energetic investment that it requires, ribosome biogenesis is closely monitored by mTOR, as is protein synthesis (30,31). Several players controlling 5′TOP translation have been identified, and here we will only mention some relevant examples. Although not by a direct interaction with the 5′TOP mRNAs, the involvement of the mTOR targets 4E-BP1 and 4E-BP2 emerged in back-to-back studies from the Sabatini and the Ruggero laboratories (29,32), showing that the pharmacological inhibition of mTOR suppresses 5′TOP translation only in 4E-BP-expressing cells. Likewise, Damgaard and colleagues demonstrated that TIA1 and TIAR mediate the translational shutdown of 5′TOPs in conditions of amino acid deprivation (33). In the same biological setting, the microRNA-10a, binding the 5′UTR downstream of the TOP element, was shown to relieve the translational inhibition by amino acid starvation (34). In the race to identify the RNA-binding protein recognizing the TOP element, LARP1, a phospho-target of mTOR (35,36,37), quickly took over the scene as it was shown by several groups to play a major role in the biology of the 5′TOP family of transcripts, even though several discrepancies arose as to whether it is a repressor or an activator of 5′TOP translation (38,39). Mechanistically, the DM15 domain of LARP1 (and its paralogue LARP1B) retains the ability to recognize and bind to the TOP element, including the 7-methylguanosine cap, with a higher affinity than eIF4E. In light of this, Lahr and colleagues have proposed a model in which LARP1 acts as the cap-binding protein of 5′TOP mRNAs (40). Irrespective of the aforementioned discrepancies, most of the studies have reported a role for LARP1 in stabilizing the 5′TOP mRNAs (39,41,42). In recent years, our group has demonstrated that stabilization of the 5′TOP mRNAs occurs through a complex of LARP1 with the 40S ribosomal subunit, which exerts an autogenous control of the ribosome biogenesis program at the translational level (42). A follow-up study has demonstrated that the 40S–LARP1–5′TOP complex is under the control of mTOR signalling with the scope of protecting the 5′TOP family from ribophagy, along with the anabolic program that this regulon encodes, particularly under unfavorable growth conditions. Importantly, the preservation of the ribosome biogenesis program in the form of mRNA can be rapidly unleashed for translation upon the reactivation of mTOR signalling, in a LARP1-dependent manner (43). In this regard, the TOP motif acts as both a protector and a coordinator of a biogenesis route involving hundreds of factors, among which the RPs must be maintained in a stoichiometric ratio. Moreover, Pan and colleagues have shown that this poised state also occurs at the level of rRNA precursors in similar biological paradigms, thus connecting RP and rRNA coordination (44). This discovery helped in rationalizing how cells can safeguard their anabolic capacity when exposed to adverse growth conditions, as observed in the tumor microenvironment or in poor metabolic niches. Despite these notions, many other aspects still need to be elucidated regarding the mTOR/40S–LARP1 axis, in light of the fact that it constitutes a heterogeneous population of 40S subunits with regulatory features that can be deregulated in pathogenic settings, as underscored by its role in 5q– syndrome (42).

## Heterogeneity in ribosomal protein composition of ribosomes: relevance in cancer

The human cytoplasmic ribosomes are composed of four rRNAs and 79 core RPs. Recent studies have shed light on the existence of ribosome heterogeneity and specialization. Ribosomes can be heterogeneous at multiple levels, which involve expression of rRNA variants, rRNA modifications, changes in the number of RPs constituting the ribosomes (substoichiometric ribosomes) or their PTMs, and even changes in ribosome-associated proteins—the ribointeractome [reviewed in (45)]. Here we discuss recent evidence highlighting how changes in RP stoichiometry and modifications have functional implications, not only in development but also in cancer progression.

### Ribosome heterogeneity and nucleolar stress: lessons from ribosomopathies

**Figure 1.**
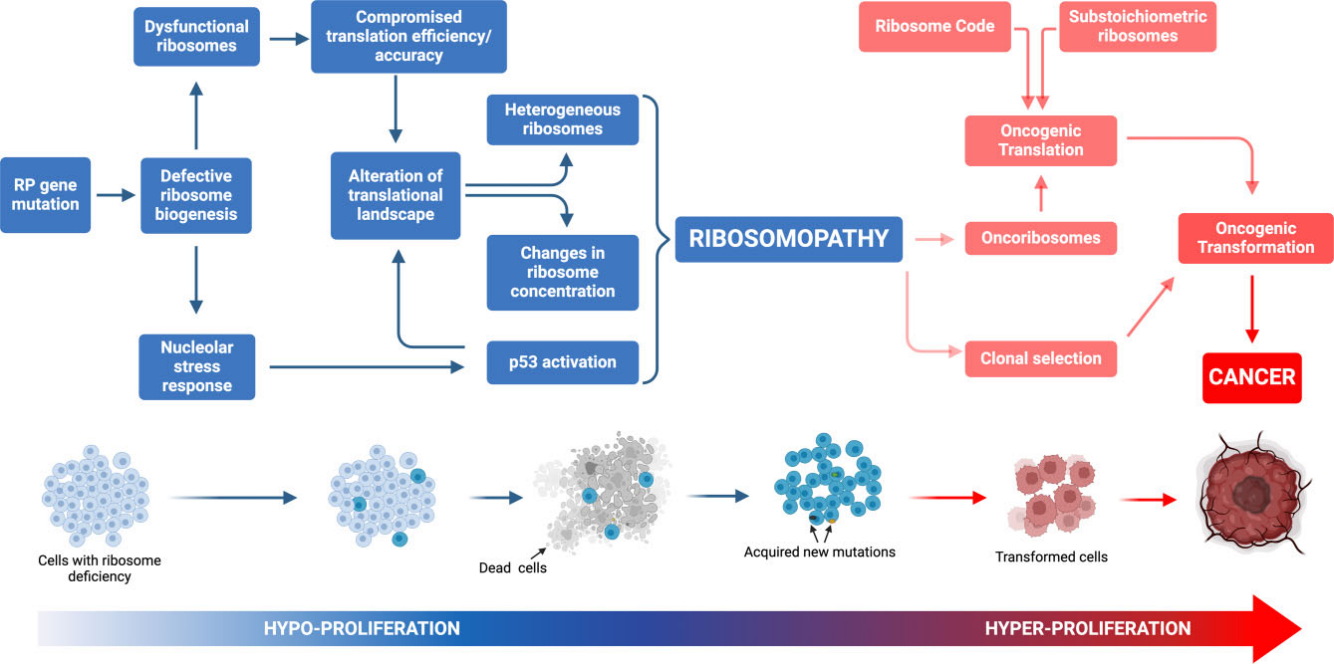
Model of cancer transformation following ribosomal protein (RP) gene mutation. RP haploinsufficient mutation(s) generally induces hypoproliferative phenotypes, exemplified by the occurrence of anaemia in ribosomopathies. Within this framework, the initial hypoproliferative state exerts selective pressure on cellular populations, facilitating the acquisition of secondary mutations that may amplify the hyperproliferative syndrome. This phenomenon culminates in the clonal proliferation of cells characterized by a modified translational profile.

Given the essential role of ribosomes, mutations in RPs or ribosome assembly factors were predicted to result in embryonic lethality. However, compelling evidence over the last decade has revealed a more nuanced reality: mutations in RPs or assembly factors lead to diseases that manifest in particular cell types, leading to tissue-specific pathologies (46,47). This contradicts the former belief that genes governing essential functions have uniform effects across tissues and cell types, necessitating a reassessment of the roles of ribosomes in health and disease. This also raises critical questions: why do alterations in ribosomes, though universally essential, lead to diseases targeting specific tissues and why do ribosomopathies, with initial proliferative deficits, show an increased risk of evolving into cancer? Several hypotheses have been proposed to explain these phenomena including: (i) the heterogeneous response to nucleolar stress; (ii) the intertissue/intercellular variability in the rate of protein synthesis; and (iii) the activation and/or repression of specific translational programs (Figure 1).

A first hypothesis for the tissue-specific effects of RP mutations suggests that the response to ribosome biogenesis impairment is not uniform, most probably due to a variable threshold of activation of p53. This mechanism is central to the pathogenesis of ribosomopathies, corroborated by diverse disease models, including those for Diamond–Blackfan anaemia (DBA), *5q–*myelodysplastic syndrome (MDS), Shwachman–Diamond syndrome (SDS), Treacher–Collins syndrome (TCS) and X-linked dyskeratosis congenita (XL-DC) (48). Impaired ribosome biogenesis checkpoint (IRBC), also called nucleolar stress response, is triggered by various alterations in ribosome biogenesis, and is a key activator of p53 in these diseases (49). The mediator of the IRBC is the pre-ribosomal complex formed by RPL5 (uL18), RPL11 (uL5) and 5S rRNA, that upon nucleolar stresses is redirected to the inhibitory binding to HDM2, leading to p53 stabilization and activation [reviewed in (50)]. Notably, deleting *TP53* mitigates many, but not all, tissue-specific phenotypes caused by RP mutations (51). For instance, craniofacial abnormalities in TCS, associated with Treacle Ribosome Biogenesis Factor 1 (TCOF1) haploinsufficiency and resulting from apoptosis of neuroepithelial and neural crest cells, can be reduced by lowering *TP53* gene dosage (52). In some DBA models, p53 inactivation also shows benefits, although this does not extend to all ribosomopathies (53,54,55), prompting the question as to whether certain cell types are inherently more susceptible to nucleolar stress (49).

Alternatively, cells with high protein synthesis requirements, consequently ‘addicted’ to ribosome activity, such as erythroid progenitors, appear to be particularly sensitive to alterations in RP expression (23). Concurrent with this hypothesis is the observation that conditions such as anaemia and bone marrow failure are prevalent features of ribosomopathies (56). Yet, this is not conclusively supported by empirical data correlating tissue-specific protein synthesis rates with the broad manifestations of RP mutations. High protein synthesis rates are characteristic of tissues such as the liver, gastrointestinal tract, muscle and skin, yet these are not universally affected by RP mutations, albeit with certain exceptions (47). This suggests that factors beyond protein synthesis demand influence the cellular sensitivity to RP mutations.

Finally, a prevalent view supported by current research argues that RP mutations contribute to alter the translational landscape (i.e. mRNAs engaged in the polysomes), due to a decrease in the number of functional ribosomes and/or the variability in ribosome composition across cell types. It is widely accepted that the reduced expression of RPs leads to defects in ribosome assembly and a concomitant reduction in their numbers. Since the ribosomes are in limiting amount and show a differential affinity for mRNAs, a reduced number of functional ribosomes would lead to a higher competition between mRNAs to be translated, known as the ‘ribosome concentration’ hypothesis. Genome-wide translational profiling in cellular models of ribosomopathies has exposed selective translation deficits, particularly affecting the transcripts of proteins involved in cell fate determination (49,56). A pronounced example is the diminished translation of GATA1, the master regulator of haematopoiesis, which contributes to erythroid hypoplasia in the bone marrow of DBA patients (57). Additionally, mRNAs containing internal ribosome entry sites (IRESs), such as the transcripts of the BCL2-associated Athano-Gene 1 (BAG1) and Cold Shock Domain-Containing E1 (CSDE1), show selective reduced translation when RPL11 (uL5) or RPS19 (eS19) levels are reduced in DBA models (58). Also, it is important to mention that chemical modifications of rRNA have been described to alter the translational output as in SDS, in which changes in rRNA pseudouridylation impair the ribosome's ability to bind IRES elements, leading to a decreased translational accuracy and a reduced translation of IRES-containing mRNAs, such as the tumor suppressor p27 and the anti-apoptotic proteins XIAP and Bcl-xL (49,59). mRNAs with extensively structured 5′UTRs, such as GATA1, are particularly vulnerable to translation inhibition following ribosome depletion (57). However, contradictory data suggest that mRNAs with short and less complex 5′UTRs, typically efficiently translated, are also sensitive to translation inhibition under conditions reducing the ribosome pools (60,61). These findings underscore the necessity to deepen our understanding of how structural and sequence mRNA regulatory elements shape translation efficiency, contributing to the distinct translational landscape observed in ribosomopathies (49,62).

Changes in the translation landscape in ribosomopathies are potentially a mixed effect of ribosome concentration and the alteration in the biosynthesis of ‘heterogeneous ribosomes’, a subpopulation of ribosomes with specific composition that are argued to favor or disfavor the translation of certain mRNAs. Studying the tissue-specific expression of RPs and their paralogues, Mauro and Edelman, among others, questioned the uniformity of ribosomes, and proposed that ribosomes may present heterogeneous compositions influencing translational dynamics and protein synthesis rates (63,64). Although challenging the traditional view of ribosomes as univariable entities, this is consistent with the fact that the deficit in certain RPs alters the spectrum of translated mRNAs without affecting the overall protein synthesis levels. For instance, the deficiency of RPL38 (eL38) impairs the translation of a subset of mRNAs encoding homeobox genes during mouse embryogenesis (65). Similarly, the reduction of RPL40 (eL40) specifically hampers the translation of vesicular stomatitis virus mRNAs in cultured human cells (66). Furthermore, the paralogs RPL3L (uL3L) and RPL10L (uL16L) are pivotal for myotube development and for the meiotic process in testes, respectively (67,68,69,70), suggesting the existence of a 'ribosomal code’ dictating how ribosomes with heterogeneous RP compositions contribute to tissue-specific functions in physiological conditions. These findings prompt a deeper exploration of the changes in the ribosomal composition, and a better understanding of their contribution to the pathogenesis of ribosomopathies and the progression to cancer.

The variability and lack of full penetrance is often observed in disorders linked to haploinsufficient RPG mutation, that may stem from compensatory mechanisms of the unaffected allele (71). In this scenario, our group discovered that in 5q– syndrome, the disrupted stoichiometric balance of RP mRNAs is a hallmark of the pathology and this is associated with the reduced expression of the RP mRNAs stabilizer LARP1, also located within the common deleted region in 5q– patients. Lowering LARP1 levels to a hemizygote gene dosage in CD34+ haematopoietic stem cells from normal donors recapitulates the features of 5q– syndrome (42). Until recently, it was thought that p53 activation and mRNA translation reprogramming were separate contributors to the clinical manifestations of ribosomopathies. Yet, a study in a DBA mouse model has revealed that Rps6 haploinsufficiency induces limb abnormalities as a result of translation deregulation. Intriguingly, *TP53* knockout reversed the translational changes due to Rps6 haploinsufficiency in most mRNAs, alleviating the phenotypic outcomes, and unveiling a novel role for p53 as a regulator of translational specificity in ribosomopathies. Mechanistically p53 up-regulates 4E-BP1, a cap-dependent translational repressor, implying a p53–4E-BP1–eIF4E axis instrumental in the selective translation changes observed (49,72). Collectively, these findings indicate that ribosome concentration, mRNA translation initiation rates and p53 activation significantly contribute to the diverse phenotypes observed in ribosomopathies.

### Onco-ribosomes and the translational programs of cancer cells: effect of RP mutations and RP paralogues on clonal evolution

The reprogramming of metabolic networks sustaining growth and proliferation is one of the hallmarks of cancer. Genetic and epitranscriptomic alterations in ribosome biogenesis are recognized regulators of cancer initiation and progression in various sporadic cancers. The oncogene *MYC* is a main activator of this process, while tumor suppressors such as *TP53, PTEN* and *RB1* have been proposed to curb this process (64,73,74,75). Ribosomopathies, initially leading to a hypoproliferative state, paradoxically culminate into an increased lifetime risk of cancer of 2.5–8.5 times, with some specific cancers such as colorectal cancer (CRC) and osteogenic sarcoma in DBA patients occurring, correspondingly, at rates 42 and 200 times higher than in the average population (56,62,76). Consistently, mutations in ribosome biogenesis factors also lead to an increased risk for various cancers (74). The haploinsufficient tumor suppressor role of RPs has also been proven in zebrafish models in which hundreds of distinct heterozygous recessive RP mutations increased cancer incidence (64,77). Addressing this paradox led to the concept of ‘onco-ribosomes’, linking the ribosome heterogeneity hypothesis to cancer progression (64,78). Also, several extraribosomal functions of RPs have been proposed to contribute to cancer progression (79). Here, we discuss a recent finding highlighting how translational rewiring associated with chronic ribosome stress provides new mechanistic insights into cancer initiation and development.

The significance of ribosome diversity during cancer progression has emerged with the concept of ‘onco-ribosomes’ (64,78). The term 'onco-ribosome' refers to ribosomes that have undergone specific alterations in cancer cells that can be either programmed—part of the cell's oncogenic adaptation processes—or result from spontaneous mutations. Programmed changes are generally driven by oncogenic signals and include the selective dysregulation and modifications of RP, as well as chemical modifications in rRNA [reviewed in (80,81)], that gear up the ribosome towards the synthesis of proteins favoring tumor progression. In contrast, spontaneous mutations may arise from genetic instability inherent in cancer cells, leading to aberrant ribosomal functions that further promote the malignant behaviour. Onco-ribosomes are predicted to differ not only in their structural make up but also in their functional dynamics across different cellular environments. This diversity has profound implications, especially in oncology, where abnormal RP expression patterns have been linked to cancer's origins and progression. Tissue-specific expression of RPs is considered as a regulatory mechanism for precise protein synthesis, mirroring the metabolic and functional demands of different tissues (82,83).

Recent studies underscore the role of RP paralogue diversity in mRNA translation and the composition of the cellular proteome (84). RP paralogues arise from gene duplications and have differentiated to perform related, yet distinct, roles. In humans, 10 RPGs form paralogous pairs with varying tissue expression. Emerging evidence supports a ‘ribosome code’, where the incorporation of RPs or their paralogues into ribosomes modulates mRNA translation specificity and cellular functions (84,85,86,87,88), notably in stress responses and cancer. For instance, up-regulation of RPL22-like1 (eL22-like 1) in CRC correlates with drug resistance, suggesting its utility as a prognostic marker and treatment response predictor. Similarly, in hepatocellular carcinoma (HCC), RPL22L1 expression associates with malignancy and drug resistance, with potential mitigation via ERK pathway inhibition (89). These findings position RP paralogues as potential targets for cancer therapy due to their tissue-specific expression and regulatory roles in disease.

The concept of substoichiometric ribosomes exposed above—those with altered RP stoichiometry—implies a role for specialized ribosomes and translational control in the rapid adaptation of the proteome of cancer cells to environmental stresses and the selective synthesis of proteins driving oncogenic transformation, survival and proliferation (90). For example, under conditions of nutrient scarcity or therapeutic pressure, alterations in the ribosome composition may favor the translation of mRNAs that support resistance mechanisms or alternative metabolic pathways. This plasticity is central for the survival and evolution of cancer cells, contributing to the heterogeneity and resilience of tumors. For instance, the RPL10-R98S mutation in T-cell acute lymphoblastic leukaemia enhances survival by favoring the translation of the anti-apoptotic protein BCL2 (91). Similarly, mutations in the SBDS gene in SDS disrupt the translation of haematopoietic regulators, leading to defective blood cell formation and increasing the risk of acute myeloid leukaemia, which is also associated with C/EBPα loss-of-function mutations (49).

A chronic deficit in ribosome biogenesis can result in the emergence of cellular subpopulations distinguished by specialized translation and/or the acquisition of RP mutations that favor survival. These mutations can activate a resistant metabolic state and skip growth controls, potentially leading to an increase in cancer aggressiveness. A thorough investigation of the differential gene expression patterns, epigenetic modifications and factors in the tumor microenvironment that contribute to onco-ribosomes will be crucial in the future. An in-depth analysis of RP expression and paralogue functions, along with the study of substoichiometric ribosomes, is vital for understanding tissue-specific disease and oncogenesis, offering a gateway to innovative cancer therapies. These therapies should aim at exploiting the distinct ribosomal configurations in cancer cells to disrupt the aberrant protein synthesis driving tumor growth, thus expanding the current cancer treatment repertoire with more precise interventions.

## RP post-translational modifications and the ribointeractome

While RP paralogs and mutations are well-documented origins of specialized ribosomes, another layer of ribosomal heterogeneity is emerging with the discovery of functional RP PTMs. Almost all possible PTMs have been identified in RPs, including methylation (lysine di- and tri-methylation), acetylation, hydroxylation (92), SUMOylation or NEDDylation (93). However, the functional consequences and biological significance of most of these modifications are still poorly understood. In this section we will discuss the functional consequeces of RP ubiquitylation, phosphorylation and UFMylation on ribosome recycling, translation fidelity and translational control.

### RP ubiquitylation

**Figure 2.**
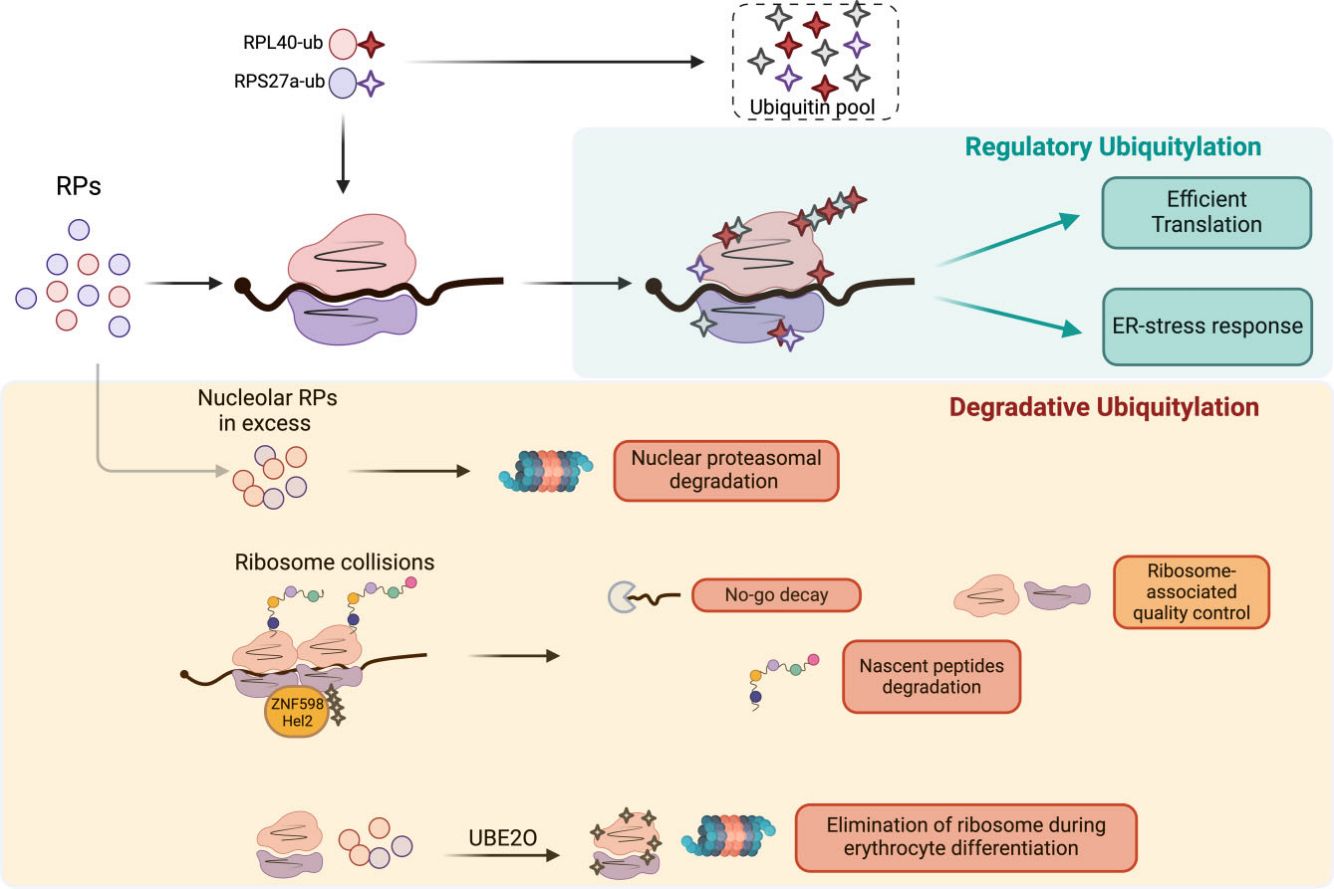
Overview of the roles of RP ubiquitylation in regulating ribosome activity or degradation. The ubiquitylation of several RPs has been proposed to regulate ribosome translation efficiency and the response to endoplasmic reticulum (ER) stress. Degradative ubiquitylation is involved in the elimination of surnumerary RPs, the resolution of collided ribosomes and the elimination of ribosomes, through UBE2O, during erythrocyte differentiation.

Ubiquitylation is probably the most studied PTM of RPs (Figure 2). It consists of the covalent modification of RPs with ubiquitin, a 76 amino acid residue peptide, either as a single molecule (monoubiquitylation) or assembled into polymeric chains (polyubiquitylation). While polyubiquitin chains linked via Lys48 largely target substrates for proteasome-mediated degradation, Lys63-linked polyubiquitin chains and monoubiquitylation are involved in non-degradation regulatory signalling (94).

In metazoans, ubiquitin is produced either as a polyubiquitin cassette (encoded by *UBB* and *UBC*) or as a fusion to two specific RPs, RPS27a (eS31) and RPL40 (eL40), from which the monomeric ubiquitin moieties are released by proteolytic cleavage. The RPS27a–Ub fusion protein is cleaved by the USP36 protease after incorporation into the ribosome, a step necessary for the maturation of the 40S subunit (95). The ubiquitin pool coming from the eL40–Ub fusion protein is essential for efficient translation in yeast (96), while in mice depletion of the eL40–Ub hybrid gene (called *UBA52*), despite having no effect on the global ubiquitin level, reduces ribosome ubiquitylation and is embryonic lethal, arguing for a regulatory role for the ubiquitylation of RPs on translation (97). Consistent with this hypothesis, Higgins *et al*. identified evolutionarily conserved, regulatory ubiquitylation sites for several RPs occurring on assembled and elongating ribosomes, during exposure to the unfolded protein response (UPR) (98). Preventing RP ubiquitylation sensitizes cells to ER stress, arguing for a protective role for ribosome ubiquitylation. This effect is potentially ascribed to a protective reduction in global protein synthesis, the specific translation of UPR genes or through an mRNA/nascent peptide quality control mechanism.

The degradative ubiquitylation pathways are important regulators of ribosome biogenesis and function. In cancer cells, RPs are produced in excess. In spite of their long half-life when incorporated in the ribosomes (>30 h), RPs are rapidly polyubiquitinated and degraded (half-life ∼6 h) by the nuclear proteasome when unassembled (99,100), with the exception of RPL5 (uL18) and RPL11 (uL5) that are mutually protected during ribosomal stress (101), or the orphan RPs, that exceed ribosome assembly during heat shock and are preserved in nucleoplasmatic condensates (102). Degradative ubiquitylation of ribosomes also proved to be important in the no-go decay (NGD) and ribosome-associated quality control (RQC) pathways induced by ribosome collisions. Secondary structures in mRNAs, lack of a STOP codon, mRNA alkylation/oxidation or amino acid starvation can all lead to ribosome stalling and ultimately collisions. When this occurs, the 40S subunits of two collided ribosomes form an interface that is recognized by the E3 ubiquitin ligase ZNF598 (Hel2 in yeast), which promotes K63-linked ubiquitylation of the ribosomal proteins RPS20 (uS10), RPS10 (eS10) and RPS3 (uS3). The ZNF598/Hel2-mediated ribosome ubiquitylation is necessary for: (i) the activation of the NGD leading to endonucleolytic cleavage of the mRNA upstream of the stalled ribosome; (ii) RQC-mediated 80S ribosome splitting and recycling; and (iii) nascent peptide degradation [reviewed in (103)]. Finally, ribosomes and RPs degradation are essential during the final stage of erythrocyte maturation, to give rise to red blood cells, mostly composed of haemoglobin (∼98% of the protein content) (104). This depends on UBE2O, an enzyme that unusually combines the functions of E2 and E3 ligase. Strikingly, UBE2O is necessary for ribosome depletion during erythrocyte differentiation, and its overexpression in HEK293 cells is sufficient to drive the elimination of individual RPs and of mature ribosomes (105). It remains to be elucidated whether UBE2O drives ribosome elimination by targeting free RPs, or also promotes ribosome disassembly. It will also be important to clarify the role of UBE2O-dependent RP ubiquitylation in other cellular contexts, and other degradative pathways such as the regulation of selective ribophagy (106).

### RP UFMylation and the ribointeractome

In a recent study, the group of Barna endogenously tagged surface-accessible RPS17 (eS17) and RPL36 (eL36), and purified the cytoplasmic ribosomes (107). Using this strategy in mouse embryonic stem cells, they identified a list of >400 ribosome-associated proteins (RAPs) independent of RNA binding or of the nascent polypeptides (RNase- and puromycin-independent). As expected, they identified actors of the protein synthesis machinery (RPs, translation factors, tRNA-related enzymes) but also novel ribosome interactors involved in mRNA translation processing and stability, rRNA and tRNA modification, cell cycle regulation, redox homeostasis and metabolism. Interestingly, a well-represented class of interactors include the protein modification enzymes (UFMylation, ubiquitylation, *O*-GlcNAcylation, acetylation and phosphorylation). Whether the interactions of these proteins with the ribosomes is involved in the modification of nascent peptides or of the translation machinery itself is intriguing.

Consistent with the latter hypothesis, Simsek *et al*. also showed that RPS3 (uS3), RPS20 (uS10) and RPL10 (uL16) are UFMylated within the 80S ribosome (107). UFMylation is the conjugation of the Ubiquitin Fold Modifier 1 (UFM1), a ubiquitin like modifier, to lysine residues by a specific conjugation machinery. UFMylation is an emerging post-translational modification, discovered in 2004, reported to be involved in ER-associated protein degradation, ribosome-associated protein quality control and ER-phagy (108). Interestingly, RPS3, RPS20 and RPL10 are in close proximity in the 80S subunit, on the solvent-exposed surface of either the 40S or 60S subunit, adjacent to the mRNA entry channel. The fact that eIF6, involved in the joining of the 40S and 60S subunits, is also UFMylated might indicate a role for UFMylation in coordinating joining of ribosomal subunits. Future studies should determine whether UFMylation also contributes to mRNA translation selectivity, and should establish the relevance of these mechanisms in other contexts. Strikingly, the genetic deletion of UFM1 conjugation enzymes results in embryonic lethality and defective erythrocyte differentiation in mice (109), a common clinical manifestation found in ribosomopathies, calling for further analyses.

### Phosphorylation of the P0/P1 stalk

Protein phosphorylation is a widespread PTM that regulates almost all cellular metabolic and signalling pathways. In mammals, striking examples of phosphorylated RPs include the phosphorylation of RPS6 (eS6), by the S6 kinases, identified 50 years ago (110), or the phosphorylation of RPL13A (UL13) in response to interferon-γ-induced inflammation, leading to its dissociation from the ribosome and to the specific translational silencing of ceruloplasmin mRNA (111). Moreover, RPS15 (uS19) can be phosphorylated by leucine-rich repeat kinase 2 (LRRK2), leading to aberrant cap-dependent and cap-independent translation, involved in the aetiology of Parkinson's disease (112). The group of Selbach analysed the phosphoproteome of the different ribosomal subcomplexes (40S and 60S subunits, the 80S subunit and polysomes) and identified 46 phosphosites in RPs spanning into 12 RPs from the small subunit and 16 RPs from the large subunit (113). They showed that amongst them, RPL12 (uL11), an evolutionarily conserved substrate of CDK1, is involved in the regulation of the translation of a specific subset of mRNA during mitosis (113).

The P-stalk is composed of RPLP0 (uL10), RPLP1 and RPLP2, which together form a pentameric complex uL10(P1–P2)_2_ on the surface of the 60S ribosome, adjacent to the A site. They are incorporated during the late stage of maturation of the 60S subunit in the cytoplasm. The P-stalk is the central element of the GTPase-associated center (GAC), whose function is to recruit elongation factors to the ribosome and stimulate their GTPase activity, which is required during each step of translation (114). The P-stalk appears to be important for translation elongation (114) and translation fidelity (115), and is required for the activation of general control nonderepressible 2 (GCN2) and the integrated stress response following ribosome stalling (116,117). The stalk proteins were named P-proteins because they are phosphorylated at their C-termini, by casein kinase CK2 and other kinases (118). Interestingly, RPLP0 can also be phosphorylated in its N-terminal domain (Y24 or T59 residues) which, in contrast to phosphorylations in the C-terminal domains, impair its binding to the 28S rRNA and the association of the P-stalk with the ribosome (119). This is particularly intriguing as only rare cases have described a dynamic association/dissociation of RPs with the ribosomes. Moreover, in *S. cerevisae*, the depletion of RPLP1/2 results in moderate changes in global translation, in contrast to RPLP0 depletion (120), but induces ribosome pausing on some transcripts encoding transmembrane domains (121). A first question to be elucidated is whether the P-stalk can be detached from the ribosome due to the phosphorylation of the solvent-exposed Y24 within uL10, or whether only P1/P2 dynamically associate with the ribosomes, as was shown in yeast (122). In either case, P-stalk PTMs and ribosome association may lead to ribosome stalling, selective translation and/or changes in the ribointeractome. Also, understanding which kinase(s) and phosphatase(s) regulate uL10 Y24/T59 phosphorylation, and whether this event is induced in stress could uncover a new layer of regulation of ribosome heterogeneity.

## Conclusions and perspectives

Protein synthesis is the final step of gene expression. Decades of research have shaped our understanding of the critical roles of ribosomal proteins as part of such a complex machinery and in the acquisition of fine-tuning mechanisms to couple its production to the metabolic supply. Mechanistic understanding of bacterial ribosomes has fostered in the clinic the design and generation of selective inhibitors of bacterial protein synthesis, used as antibiotics. On the other hand, over the last decade, many pieces of evidence have shown that in humans the ribosome composition is not uniform across tissues and developmental stages, revealing a striking adaptability of the cellular translational program to internal and external cues. Tumor cells utilize these regulatory mechanisms to better adapt to unfavorable growth conditions and hence for clonal evolution. Considerable progress has established an unprecedented level of complexity with the discovery of the onco-ribosomes, the substoichiometric ribosomes and the description of PTMs in RPs, associated with alterations in ribosome dynamics and in the cancer translational landscapes. Decoding ribosome complexity, with a systematic approach, is a pivotal challenge of current research which promises to have a clinical impact both for ribosomopathies and for cancer treatment.

## Data Availability

No new data were generated or analyzed in support of this research.
